# Endocrine and surgical outcomes after medial cavernous sinus wall resection for functioning pituitary adenomas: a systematic review and meta-analysis

**DOI:** 10.1007/s10143-026-04502-8

**Published:** 2026-09-17

**Authors:** Luis O. Vargas, Vratko Himic, Vagif Kazimli, Adonicah Cummings, Daniel Kreatsoulas, Arman Jahangiri, Daniel M. Aaronson, Ashish H. Shah, Ricardo J. Komotar, Michael E. Ivan, Zachary C. Gersey

**Affiliations:** 1 Department of Neurosurgery, University of Buffalo Jacobs School of Medicine and Biomedical Sciences, Buffalo, NY USA; 2 Department of Neurological Surgery, University of Miami Miller School of Medicine, Miami, FL USA; 3 Division of Neurological Surgery, Saint Louis University School of Medicine, St. Louis, MO USA; 4 Department of Neurosurgery, Loma Linda University Medical Center, Loma Linda, CA USA

**Keywords:** Functioning pituitary adenoma, Medial cavernous sinus wall resection, Endoscopic endonasal surgery, Biochemical remission, Gross total resection, Skull base surgery

## Abstract

Functioning pituitary adenomas (FPAs) require both cytoreduction and durable endocrinological remission (ER), which is often limited by occult invasion of the medial cavernous sinus wall. Selective medial cavernous sinus wall resection (MCSWR) has emerged as a targeted approach, but its endocrine benefits and safety profile remain incompletely understood. We synthesize syndrome-specific remission, resection, recurrence, and complication rates after MCSWR in adults with FPAs. We conducted a systematic review and meta-analysis of PubMed, Scopus, and Embase through 2025, including adults with GH-, ACTH-, and prolactin-secreting adenomas who underwent intentional medial cavernous sinus wall resection as described by the original studies and had at least one postoperative endocrine outcome. Two reviewers extracted study- and tumor-level data on resection, ER, recurrence, and complications. Pooled event rates were estimated with random-effects models. Thirteen studies (11 retrospective, 2 prospective) involving 493 patients met inclusion criteria. Among 344 patients with available endocrine follow-up, pooled ER was 85% (95% CI:77–91%), and gross total resection (GTR) was 92% (95% CI:81–97%). Recurrence occurred in 6% (95% CI:3–13%). Subgroup ER rates were 87% for acromegaly, 89% for Cushing’s disease, and 78% for prolactinomas, with corresponding GTR rates of 95%, 91%, and 80%. Complications were rare: ICA injury 0%, cranial nerve III and VI palsies 3% each, and postoperative CSF leak 4%. Most studies had a serious or critical risk of bias, mainly due to confounding and single-arm designs. Follow-up ranged from 9 to 30 months in contributing cohorts, with earlier series reporting ranges up to 1–49 months. MCSWR was associated with high reported ER and GTR rates, low reported recurrence, and acceptable morbidity in carefully selected patients with FPAs, particularly somatotroph and corticotroph tumors. However, the evidence mainly comes from high-volume centers and non-comparative series with limited follow-up, highlighting the need for prospective, multicenter studies with standardized endocrine endpoints, histological confirmation, and long-term follow-up.

## Introduction

Pituitary adenomas (PAs) are predominantly benign tumors of the sellar region originating from the adenohypophysis [1, 2]. Functioning pituitary adenomas (FPAs), including those causing acromegaly, Cushing’s disease (CD), and hyperprolactinemia [3], pose a unique therapeutic challenge: unlike non-functioning tumors, the goal is two-fold; to achieve cytoreduction as well as endocrinological remission (ER) to prevent systemic sequelae [4]. While transsphenoidal surgery (TSS) is still the primary treatment, outcomes have sometimes been limited by cavernous sinus invasion, as reflected in the Knosp classification. When tumors extend laterally, standard extracapsular dissection often fails to normalize hormone levels, resulting in ongoing metabolic issues and higher mortality [1].

The main anatomical barrier to cure in these invasive cases is the medial cavernous sinus wall (MCSW). The extent of invasion is traditionally assessed using the Knosp grading system on coronal MRI [5], where higher grades (3 and 4) strongly predict complex resections and lower rates of gross total removal [6]. Therefore, there is generally a positive correlation between Knosp grade and the risk of tumor recurrence, as the MCSW often serves as a sanctuary site for microscopic disease that remains after standard surgery [7]. Since the MCSW is susceptible to tumor invasion due to its proximity to the pituitary gland, involvement of this area significantly increases the likelihood of disease progression [8].

Historically, Jules Hardy’s development of transsphenoidal selective adenomectomy helped establish the modern microsurgical treatment of pituitary adenomas; however, the cavernous sinus was generally considered a strict anatomical boundary, and lateral exploration was avoided because of the risk of neurovascular morbidity [9]. The surgical paradigm later evolved following Buchfelder and Fahlbusch’s 1988 analysis of 147 transsphenoidal operations for parasellar adenomas, which showed that transsphenoidal exploration could be feasible in selected cases, particularly when there was focal cavernous sinus invasion [10]. This experience laid the groundwork for more targeted lateral approaches. Subsequently, Knosp et al. established a standardized MRI-based framework for classifying cavernous sinus invasion, enabling more precise preoperative stratification [5]. Together, this progression from avoidance to selective exploration and radiographic classification helped shape modern anatomically guided endoscopic approaches. Today, improved visualization, neuronavigation, Doppler ultrasonography, and selective use of intraoperative MRI have renewed interest in targeted resection of the medial cavernous sinus wall for carefully selected functioning adenomas [12, 19].

To address this, selective endoscopic resection of the MCSW has become a viable strategy to detect occult invasion. Used by select high-volume skull-base centers [11], this technique targets the dural substrate of the disease. Early single-center experience by Cohen-Cohen et al. (2018) reported complete tumor resection and about 97% biochemical remission among functioning adenomas (growth hormone–, prolactin–, and ACTH-secreting tumors) following selective medial cavernous sinus wall resection, without injury to the internal carotid artery [12]. Later meta-analyses, such as the 2023 study by Pontes et al., reported pooled remission and gross total resection (GTR) rates of about 63% and 73%, respectively, with minimal carotid injury [13]. These results suggest that medial cavernous sinus wall resection (MCSWR) may be a feasible adjunct, but the actual benefit for functioning tumors remains hard to measure because most large reviews include non-functioning cases.

The current literature has not fully isolated the impact of this technique on endocrine outcomes. While systematic reviews by de Macêdo Filho et al. [8] and Pontes et al. [13] have evaluated the safety of the approach, these analyses mainly combined functioning and non-functioning adenomas and focused primarily on technical feasibility and complication profiles. As a result, the endocrinological efficacy of medial wall resection in functioning tumors remains uncertain, especially regarding whether MCSWR is associated with improved ER compared with traditional TSS without medial wall resection, and whether any benefits occur without increasing the risk to vascular or cranial nerve morbidity. Clarifying these syndrome-specific outcomes is crucial for endocrinologists and neurosurgeons in determining when the potential for improved endocrine outcomes may justify selective cavernous sinus exploration.

For the purposes of this review, we defined MCSWR as intentional medial cavernous sinus wall resection as described by the original studies, distinct from simple debulking of visible tumor. Building on prior series, we conducted a comprehensive, PRISMA-conforming systematic review and meta-analysis focused solely on FPAs undergoing MCSWR. Our main goal was to determine pooled, syndrome-specific rates of ER. We also aimed to evaluate the reported safety profile of this technique, including vascular and cranial nerve morbidity.

## Materials and methods

### Study selection

We conducted a systematic review and meta-analysis in accordance with PRISMA guidelines. PubMed, Scopus, and Embase were searched from inception through March 24, 2025, using predefined MeSH/Emtree terms and keywords related to “pituitary adenoma,” “cavernous sinus,” and “medial wall resection.” The reference lists of included articles and relevant reviews were manually screened for additional studies through forward and backward reference review. The review protocol was registered prospectively on PROSPERO (CRD420251020234).

### Eligibility criteria

We included clinical studies of any design that met these criteria: (1) adults with FPAs (GH-, ACTH-, prolactin-secreting); (2) intentional medial cavernous sinus wall resection as described by the original authors, rather than simple debulking of visible tumor; (3) reporting at least one postoperative endocrine outcome based on biochemical criteria; and (4) sample size greater than three patients. We excluded non-English articles, conference abstracts lacking extractable outcome data, and overlapping cohorts, retaining the most comprehensive, non-overlapping reports. For mixed cohorts that included both functioning and non-functioning adenomas, studies were only eligible if outcomes for functioning tumors were reported separately; in such cases, we extracted data exclusively from the functioning subgroups. Studies where the operative description did not clearly distinguish medial wall resection from standard transsphenoidal debulking were excluded to prevent misclassifying conventional surgery as MCSWR.

### Screening and data extraction

After removing duplicates, titles and abstracts were screened, followed by a full-text review of potentially eligible studies. Two reviewers independently extracted data into a standardized spreadsheet, including study design, setting, sample size, demographic characteristics, follow-up duration, operative approach, functional subtype, syndrome-specific remission counts, and complications. Disagreements were resolved by consensus, with arbitration by a third reviewer when needed.

### Endpoints of interest

The primary endpoint was ER, defined by study-specific biochemical criteria aligned with current endocrine standards (for example, normalized age- and sex-adjusted IGF-1 with appropriate GH suppression in acromegaly; ER of hypercortisolism in CD; and normalization of prolactin levels off dopamine-agonist therapy in prolactinoma). Secondary endpoints included GTR, as determined by the surgeon’s intraoperative assessment and, when available, early postoperative MRI; recurrence or persistence requiring additional therapy or reoperation; and perioperative complications, such as internal carotid artery (ICA) injury, cranial nerve III and VI palsy, postoperative cerebrospinal fluid (CSF) leak, and diabetes insipidus (DI). CSF leak was recorded only when occurring postoperatively.

### Subgroup definitions

Prespecified subgroup analyses assessed outcomes by functional subtype (GH-, ACTH-, and prolactin-secreting tumors) and tumor size (macroadenoma versus microadenoma), when available. Studies reporting only non-stratified outcomes were analyzed separately and were not pooled with subtype-specific models.

### Statistical analysis

All statistical analyses were conducted using R (version 4.x) with the *meta* package. For each primary and secondary endpoint, we calculated pooled proportions and 95% confidence intervals using the *metaprop* function with inverse-variance weighting and a *logit* transformation (sm="PLOGIT”) to stabilize variance, especially for proportions near 0 or 1. A continuity correction of 0.5 was applied to studies with zero cell counts to facilitate the calculation of logit-transformed proportions. Both fixed-effect and random-effects models were created; the random-effects models, using the DerSimonian–Laird estimator for between-study variance (τ²), were prespecified as the primary models and are reported throughout the manuscript.

Between-study heterogeneity was assessed using the I² statistic and Cochran’s Q test, with I² values greater than 50% interpreted as indicating substantial heterogeneity. To prevent double-counting, overall pooled analyses (ER, GTR, and complications) were conducted on a deduplicated dataset, in which, for each cohort or institution, only the most comprehensive, non-overlapping report within a specific time frame was included. Subgroup models (such as GH-only, ACTH-only, or prolactinoma-only cohorts) were run on filtered datasets restricted to the relevant functional subtype and analyzed separately from the overall pooled models.

Given heterogeneity in endocrine assays and biochemical cutoffs across centers, we accepted each study’s author-defined criteria for remission, provided they aligned with current consensus standards for that syndrome. Because medial wall involvement was inconsistently characterized using radiographic (e.g., Knosp classification), intraoperative, or histologic criteria, we did not quantitatively pool invasion grades. Instead, we recorded the grading system used in each study and summarized these patterns qualitatively, while treating all included cases as intentional medial wall resections for outcome pooling.

For safety outcomes with low expected incidence (ICA injury, cranial nerve palsy, CSF leak), we included only studies in each pooled analysis that explicitly reported whether the specific complication was present or absent. Studies that did not clearly specify if a particular complication occurred were excluded from that outcome-specific model.

Risk of bias was assessed for each included study using the ROBINS-I framework for non-randomized interventions, and the results were displayed graphically using the ROBVIS tool. Two reviewers independently evaluated each domain, and disagreements were resolved by consensus with a third independent reviewer.

## Results

### Baseline characteristics of all studies

**Fig. 1:**
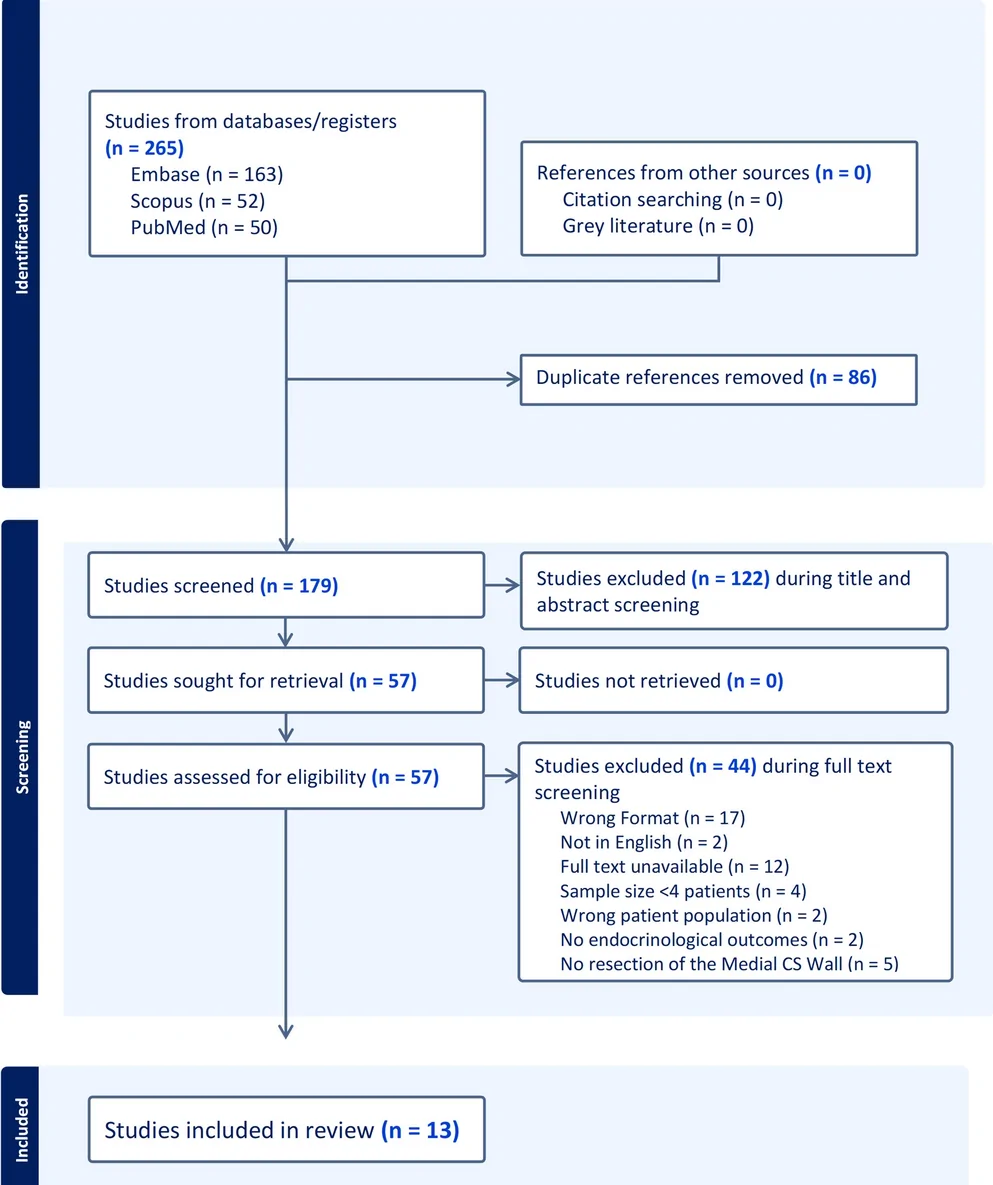
PRISMA flowchart showing study selection

The initial database search identified 265 records in PubMed (50), Scopus (52), and Embase (163) (Fig. 1). After removing duplicates (*n* = 86), 179 titles and abstracts were screened, and 57 full-text articles were assessed for eligibility. Thirteen studies met all inclusion criteria and were included in the qualitative and quantitative analyses. Of these, 11 were retrospective cohorts and 2 were prospective single-center series [11, 14]. The studies were conducted across the USA, China, Japan, and Canada. Overall, 493 patients underwent MCSW resection for FPAs, including acromegaly, CD, and prolactinomas. Reported follow-up per cohort ranged from 9 to 30 months for studies that provided a single mean or median value, with means between 9 and 15.6 months, and from 1 to 49 months and 11–34 months in the two older series that reported follow-up only as ranges. Three studies did not report follow-up duration.

Although 13 studies were included in the overall synthesis, the number of studies contributing to each quantitative endpoint varied depending on how outcomes were reported (Table 1). For the primary outcome of ER, extractable postoperative endocrine data were available from 10 studies (344 patients). GTR rates suitable for pooling were available from 5 studies (99 patients), and recurrence data were available from 6 studies (111 patients). For syndrome-specific analyses, GH-secreting adenomas were described in 10 studies (257 patients), ACTH-secreting tumors in 6 studies (50 patients), and prolactinomas in 5 studies (35 patients). Subtype-specific GTR could be extracted from 4 studies for GH-secreting tumors (38 patients), 4 for ACTH-secreting tumors (21 patients), and 3 for prolactinomas (35 patients). Denominators for complication analyses also varied by outcome, reflecting that only studies explicitly reporting a given complication were included in that safety model.

**Table 1:** Baseline characteristics of the 13 included studies, summarizing study design, patient demographics, tumor subtypes, and follow-up duration

Study	Country	Study design	Age (yrs)	Patients total (*N*)	Females	Follow-up, months	Tumor type (and number)
Tian L et al. 2025 [31]	China	Retrospective	44.9 (mean)	56	35	9 (mean)	Acromegaly
Oberman et al. 2025 [11]	USA	Prospective	49.5 (median)	20	13	12 (median)	Acromegaly 8, ACTH 6, Prolactinoma 6,
Liang et al. 2024 [32]	China	Retrospective	45 (mean)	20	9	12 (mean)	GH 12, ACTH 2, Prolactinoma 6
Liang et al. 2024 [33]	China	Retrospective	45 (mean)	12	NA	12 (mean)	GH 12
Ishida et al. 2022 [15]	Japan	Retrospective	48.7 (mean)	102	64	NR	GH 67, ACTH 23, Prolactinoma 10
Mohyeldin et al. 2022 [14]	USA	Prospective	50 (mean)	50	NA	15.56 (mean)	Acromegaly 21, ACTH 9, Nonfunctioning 20
Cohen-Cohen et al. 2018 [12]	USA	Retrospective	49 (mean)	50	25	30 (mean)	Acromegaly 16, ACTH 9, Prolactinoma 10
Sanchez et al. 2024 [34]	USA	Retrospective	NA	14	NA	NR	GH 4, ACTH 5, Prolactinoma 4
Mohyeldin et al. 2021 [21]	USA	Retrospective	NA	48	NA	NR	NA
Omar et al. 2020 [35]	Canada	Retrospective	40.9 (mean)	16	13	11 (median)	GH 6, ACTH 5, Prolactinoma 5
Nagata et al. 2019 [36]	Japan	Retrospective	57.9 (mean)	14	7	11.8 (mean)	GH 12, ACTH 2
Woodworth et al. 2014 [37]	USA	Retrospective	47.3 (mean)	36	22	1–49 (range)	GH 10, ACTH 3, Prolactinoma 7, Gonadotropic 2
Nishioka et al. 2014 [38]	Japan	Retrospective	NA	55	NA	11–34 (range)	Acromegaly 55

Follow-up is shown as mean or median as reported in the original article; when only a range was provided, it is shown as ‘range’. *NR -* not reported

### Pooled analysis of ER and GTR

**Fig. 2:**
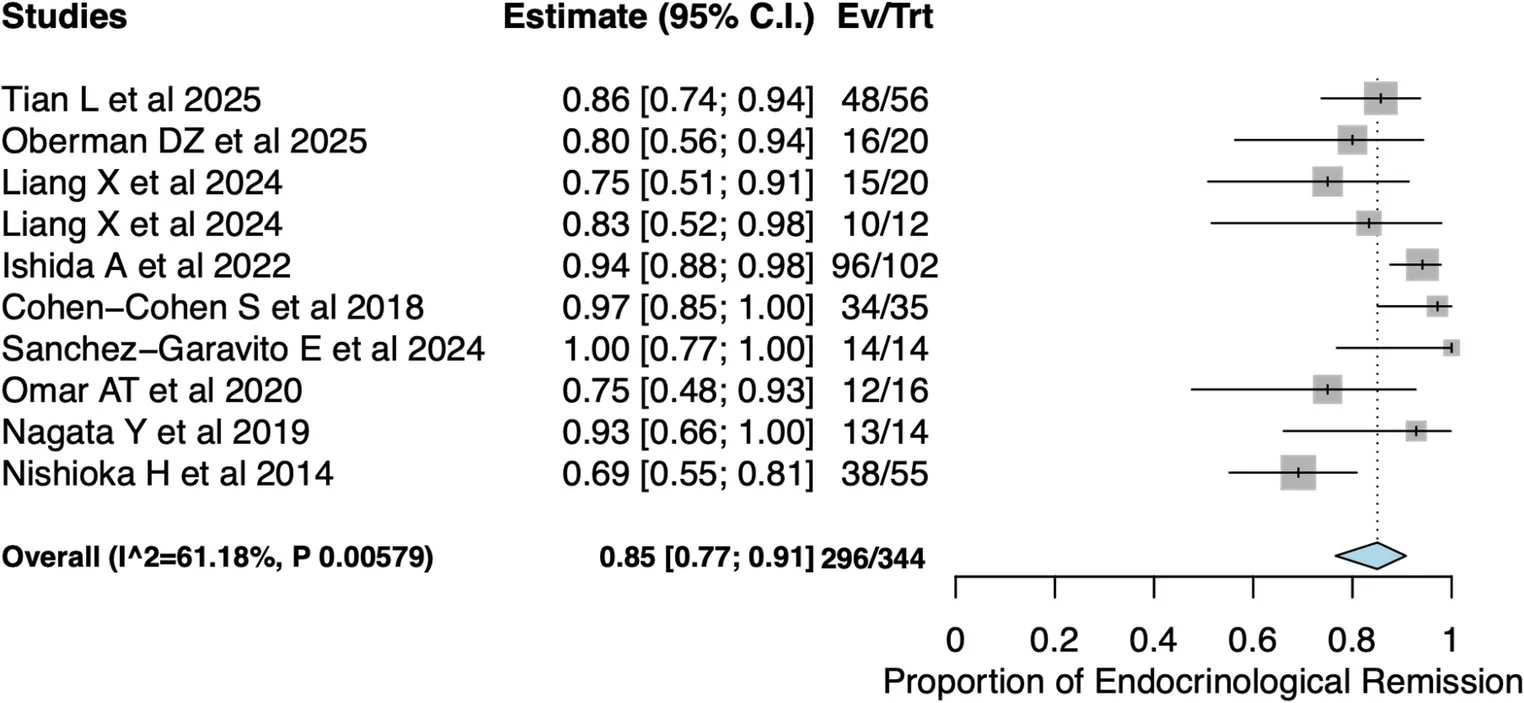
Forest plot of endocrinological remission (ER) across all studies, showing a pooled proportion of 85% (95% CI 0.77–0.91)

**Fig. 3:**
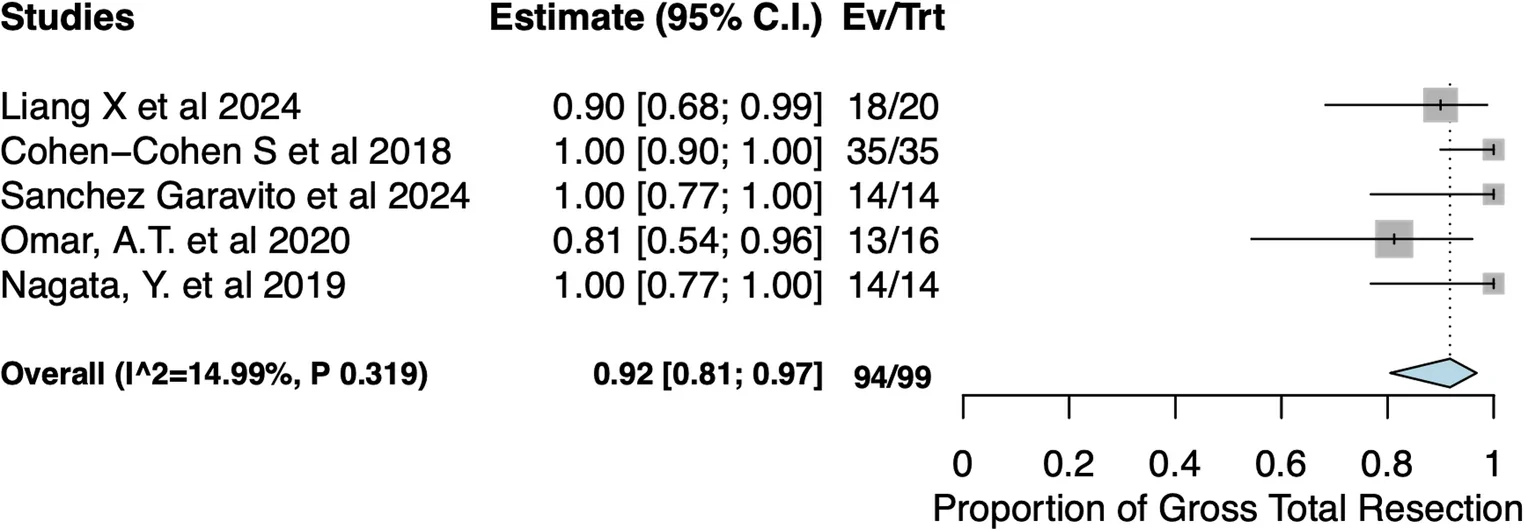
Forest plot of gross total resection (GTR), demonstrating a pooled resection rate of 92% (95% CI 0.81–0.97)

**Fig. 4:**
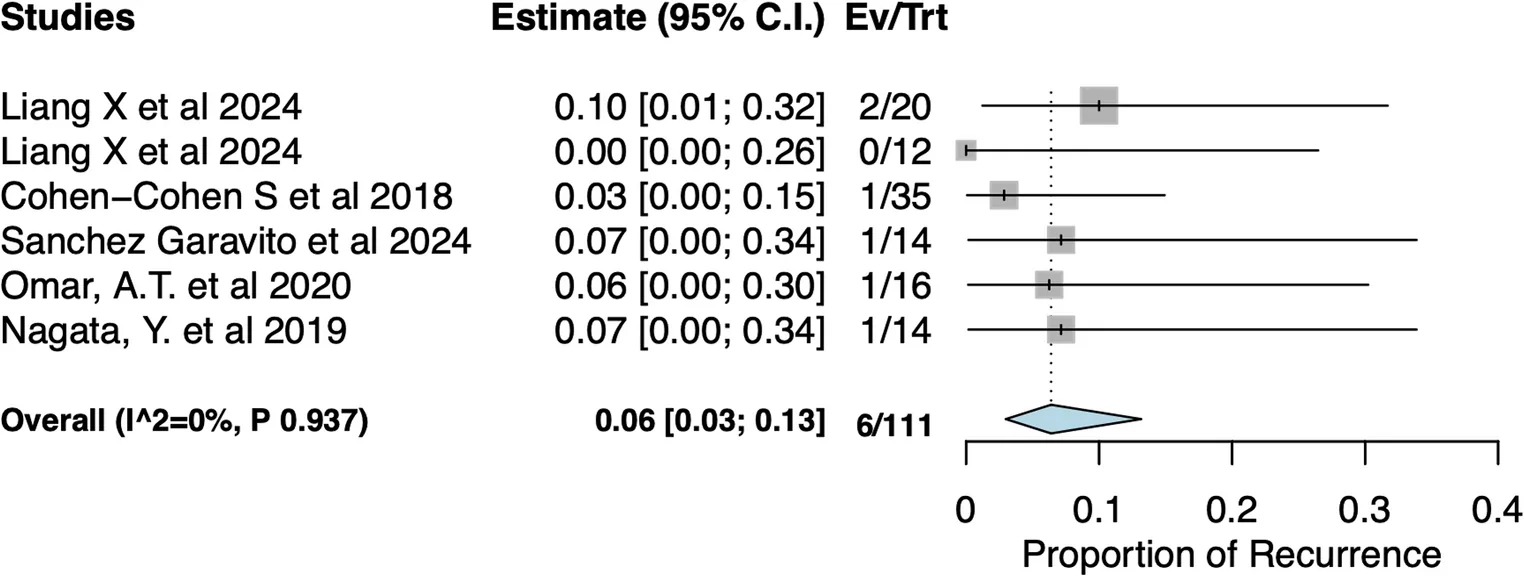
Forest plot of tumor recurrence, indicating a pooled recurrence rate of 6% (95% CI 0.03–0.13)

The primary outcome of our systematic review was endocrinological remission (ER). Among 344 patients with extractable endocrine follow-up data, the pooled ER proportion was 85% (95% CI 77–91%; 296/344) (Fig. 2). The secondary outcome was GTR; across 5 studies, 94 of 99 patients achieved GTR, resulting in a pooled proportion of 92% (95% CI 81–97%) (Fig. 3). Another outcome of interest was recurrence. Six studies reported recurrence in 6 of 111 patients, corresponding to a pooled proportion of 6% (95% CI 3–13%; Fig. 4). Among the cohorts contributing to these endocrine outcomes, study‑level follow‑up ranged from 9 to 30 months in series reporting a single mean or median value, with earlier series reporting follow‑up only as ranges (1–49 months and 11–34 months).

### Subgroup analysis

**Fig. 5:**
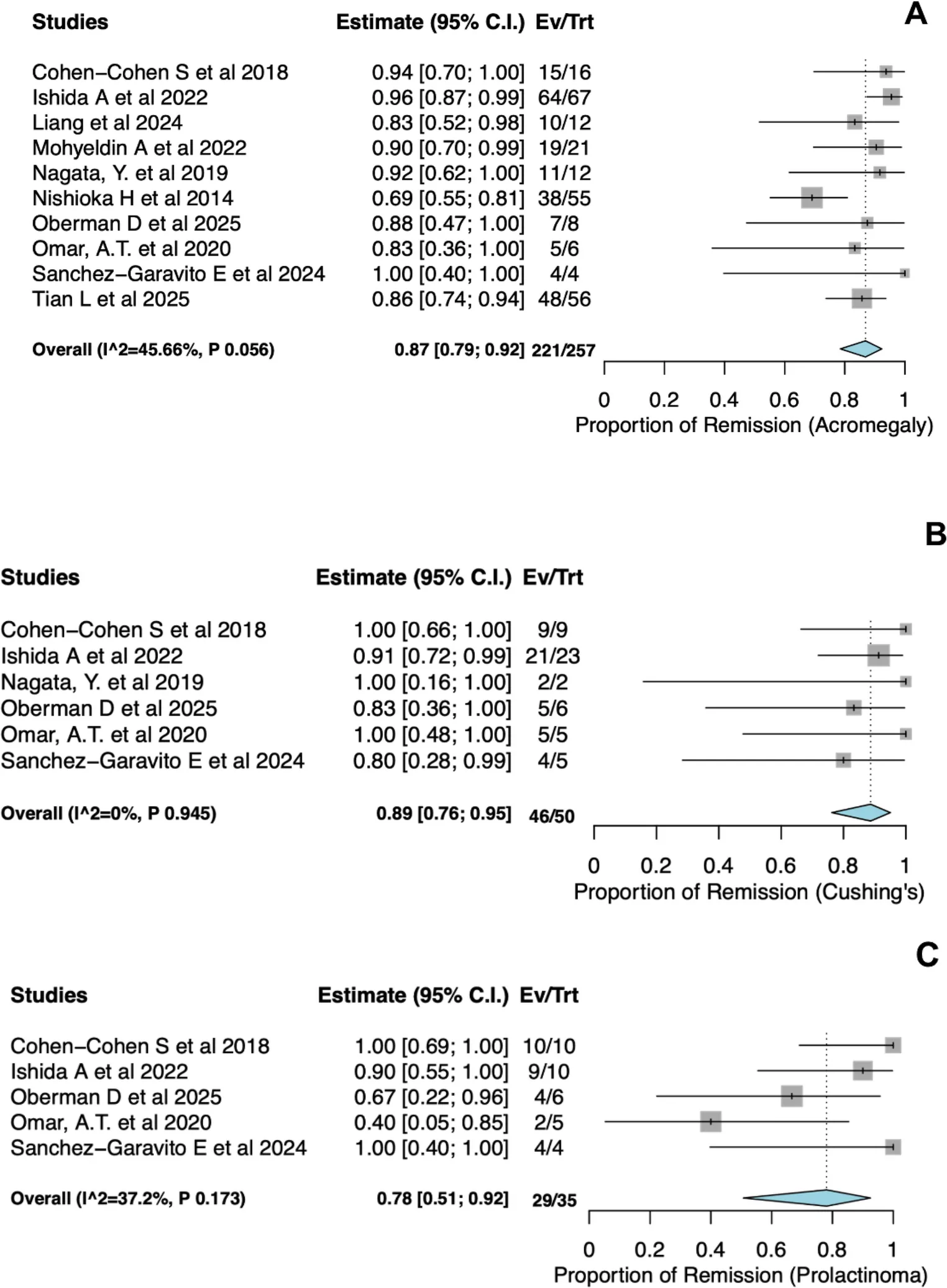
Subgroup analysis of endocrinological remission by tumor subtype: (**A**) Acromegaly (87%), (**B**) Cushing’s disease (89%), and (**C**) Prolactinoma (78%)

A subgroup analysis was conducted to determine whether outcomes varied by pituitary tumor type. In this analysis, GH-secreting tumors were the most commonly reported subgroup with respect to ER. Ten studies, including 257 patients, were combined; ER was reported in 221 patients, resulting in a pooled proportion of 87% (95% CI 79–92%) (Fig. 5A). ACTH-secreting tumors had an ER rate of 89% (95% CI 76–95%) across six studies, involving 46 of 50 patients (Fig. 5B). Finally, for prolactinomas, five studies with 29 out of 35 patients showed an ER proportion of 78% (95% CI 51–92%) (Fig. 5C). Among studies that reported subtype-specific follow-up, acromegaly cohorts had study-level follow-up ranging from 9 to 22 months, Cushing’s disease cohorts from 11 to 24 months, and prolactinoma cohorts from 12 to 28 months.

**Fig. 6:**
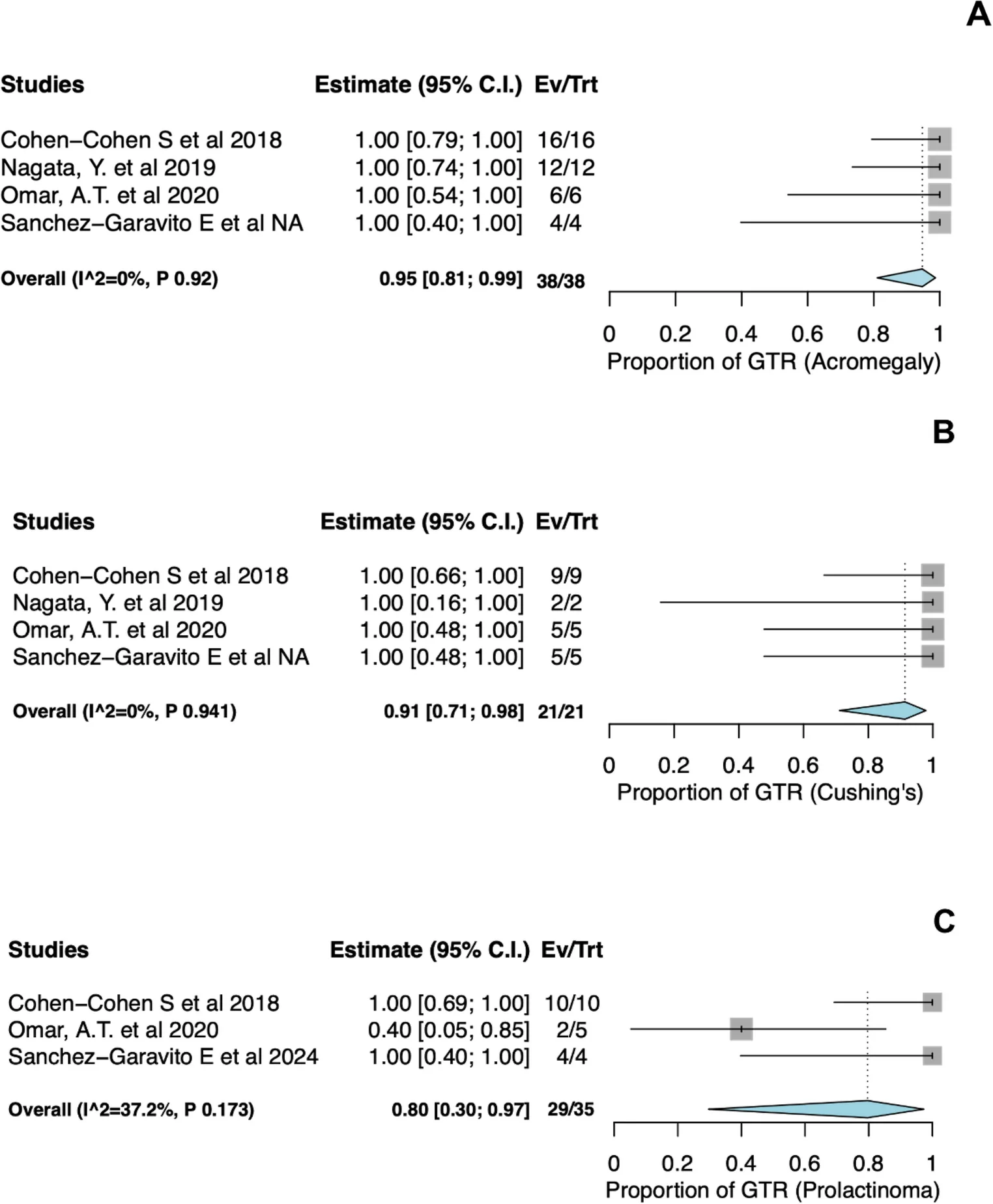
Subgroup analysis of gross total resection (GTR) by tumor subtype: (**A**) Acromegaly (95%), (**B**) Cushing’s disease (91%), and (**C**) Prolactinoma (80%)

The second endpoint of interest for subgroup analysis was GTR. Among GH-producing tumors, four studies including 38 patients were combined, with 38 achieving GTR and an overall proportion of 95% (95% CI 81–99%) (Fig. 6A). In ACTH-producing tumors, four studies reported that all 21 patients achieved GTR, resulting in a pooled proportion of 91% (95% CI 71–98%) (Fig. 6B). Three studies reported prolactinoma-specific GTR, with 29 of 35 achieving GTR (80%; 95% CI 30–97%) (Fig. 6C).

### Complications

**Fig. 7:**
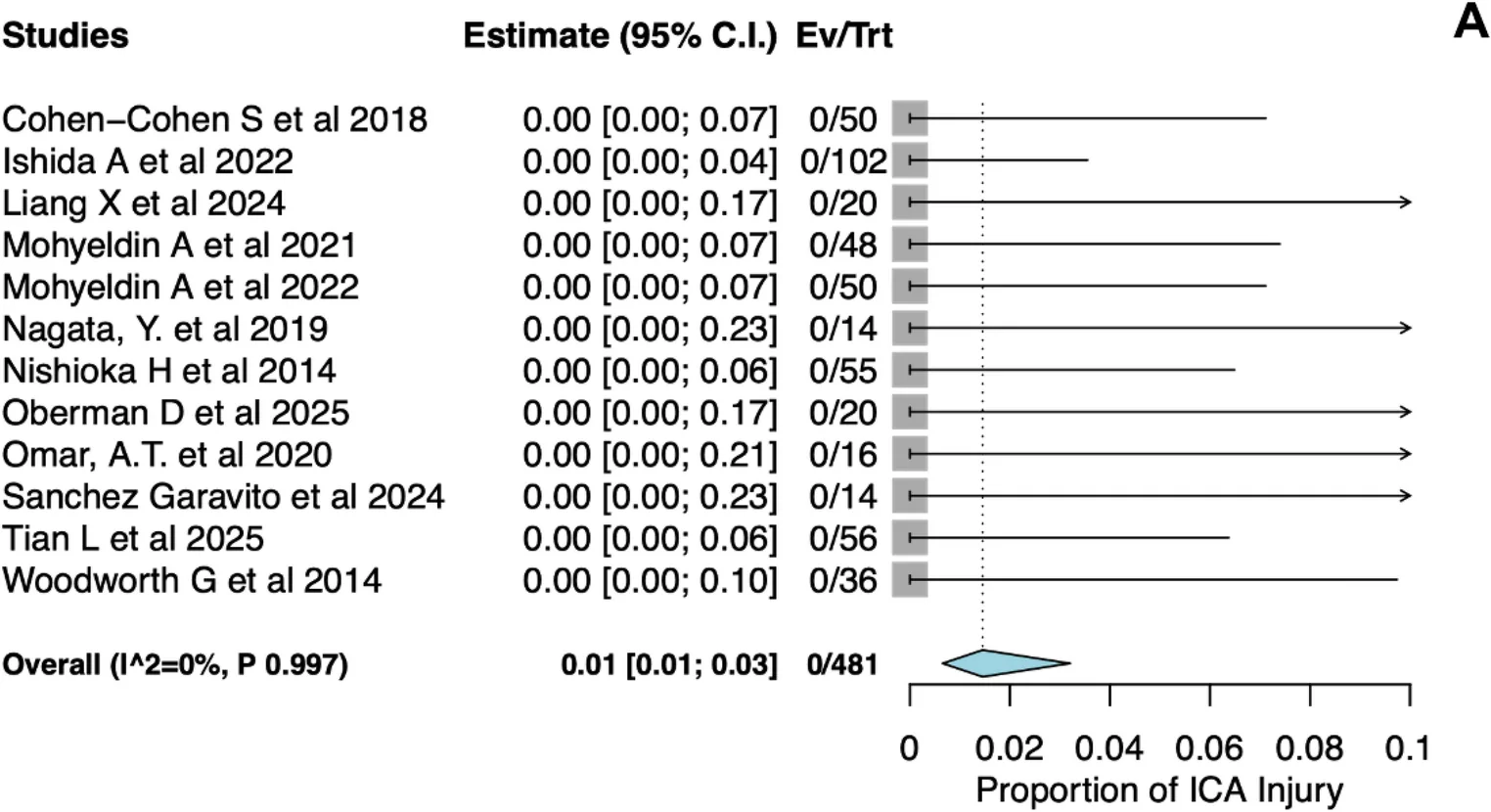
Forest plots of vascular complications showing (**A**) a 0% rate of internal carotid artery (ICA) injury

**Fig. 8:**
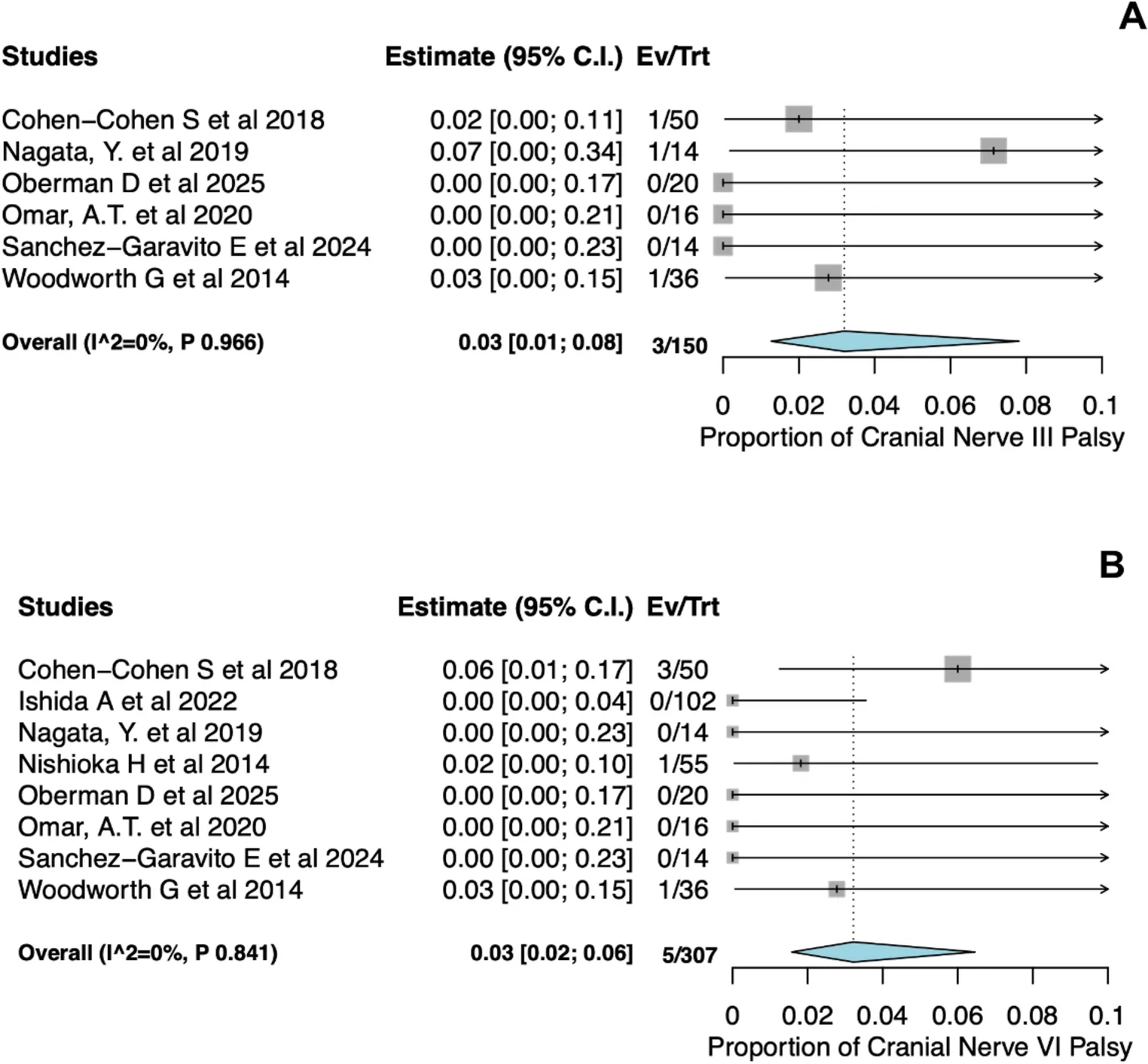
Forest plots of cranial nerve morbidity showing (**A**) a 3% rate of cranial nerve III palsy and (**B**) a 3% rate of cranial nerve VI palsy

**Fig. 9:**
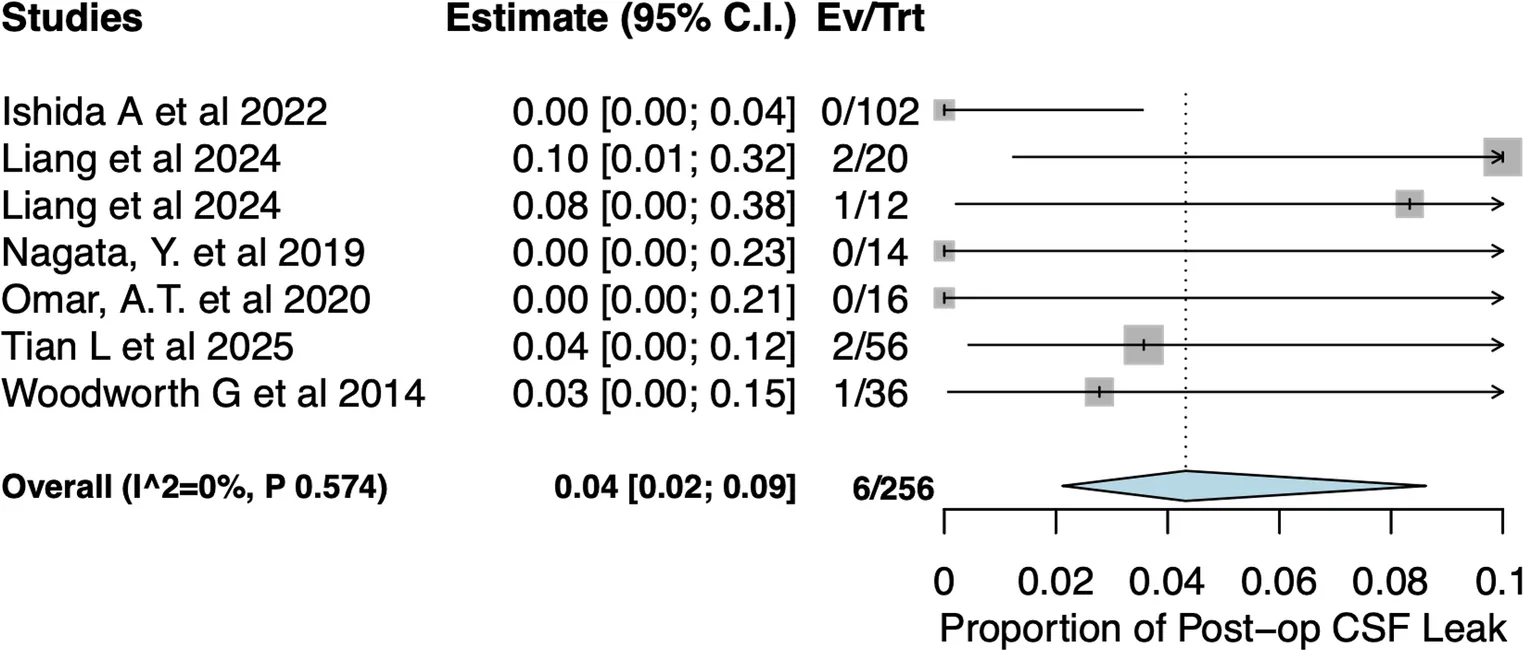
Forest plot of postoperative CSF leak, showing a pooled complication rate of 4% (95% CI 0.02–0.09)

An important aspect of this approach is the potential for complications. The risk of ICA injury was rare, occurring in 0 of 481 patients (95% CI 0.1–1.0%) (Fig. 7A). Cranial nerve III palsy occurred in 3 of 150 patients, resulting in a pooled proportion of 3% (95% CI 1.0–8.0%) (Fig. 8A), while sixth cranial nerve palsy was observed in 5 of 307 patients, resulting in a pooled proportion of 3.0% (95% CI 2.0–6.0%) (Fig. 8B). Lastly, postoperative CSF leak occurred in 6 of 256 patients, for a pooled proportion of 4% (95% CI 2.0–9.0%) (Fig. 9).

### Risk of bias

Risk-of-bias assessment using ROBINS-I showed that most studies had a serious or critical risk of bias, mainly due to confounding and the lack of prospective control groups (Supplementary Fig. 1).

## Discussion

This systematic review and meta-analysis summarize the outcomes of MCSWR for FPAs across 493 patients. The pooled ER rate was 85%, along with a GTR rate of 92% and a low recurrence rate of 6% among studies with extractable follow-up data. Reported complications, including cranial nerve III and VI palsy (3% each), postoperative CSF leak (4%), and no reported ICA injuries, were infrequent. However, these findings should be interpreted cautiously because the available evidence is largely derived from retrospective, single-arm series performed at high-volume pituitary and skull base centers. Therefore, although MCSWR appears to achieve high biochemical control with an acceptable safety profile in carefully selected patients, the current evidence does not establish superiority over standard transsphenoidal surgery or isolate the independent effect of medial wall resection from surgeon experience, institutional expertise, and patient selection.

The high remission rate observed in this meta-analysis probably reflects MCSWR’s ability to eliminate microscopic disease within the MCSW, a compartment repeatedly shown to contain occult invasion [15]. Histologic studies have shown that the medial wall is very thin, averaging 0.195 mm (range 0.080–0.387 mm), and it is often infiltrated even when it appears normal intraoperatively [16]. Ishida et al. confirmed medial wall invasion in 57% of cases in which it was not suspected during surgery, underscoring why standard transsphenoidal resection often leaves hormonally active tissue cells behind [15]. This may represent a potential benefit of MCSWR: narrowing the gap between radiographic GTR and biochemical remission, whereas standard surgery for invasive functioning adenomas usually achieves remission in only 20–40% of patients [17, 18]. This discrepancy suggests that standard GTR rates are frequently overcalled, as microscopic invasion of the medial wall remains invisible even under high-definition endoscopy and MRI. By contrast, our meta-analysis shows that MCSWR achieved a pooled GTR of 92% and an ER of 85%, supporting the idea that selectively resecting the medial wall when occult involvement is suspected addresses this invisible microscopic invasion and may contribute to improved biochemical remission in selected patients. Furthermore, the technique aligns with anatomical studies that identify the medial wall as a distinct, resectable dural layer rather than an extension of the pituitary capsule, allowing for safe extracapsular dissection [19]. These findings support the idea that the medial wall is a crucial substrate for biochemical persistence and that resecting it may contribute to biochemical remission in selected cases.

Across tumor subtypes, the remission benefits of MCSWR were consistent but varied in magnitude. In acromegaly, the pooled remission rate was 87%, which substantially exceeds historical benchmarks for invasive somatotroph adenomas, where standard surgery usually achieves around 30% remission in Knosp 3–4 tumors [17, 18]. This improvement aligns with studies indicating that GH-secreting tumors have a strong tendency to invade the medial wall, making them especially suitable for wall resection [20]. Corticotroph tumors showed the highest remission rate (89%) compared to traditional outcomes of 30–58% for invasive Cushing’s disease, where microscopic dural invasion often results in persistent hypercortisolism [21]. Prolactinomas had a lower remission rate (78%) than other subgroups, but it remained notably higher than the approximately 38% remission rate reported in surgically treated dopamine-agonist-failed prolactinoma patients [22]. The durability of remission across all three subtypes, combined with high GTR rates (80–95%), supports the idea that medial wall removal effectively eliminates an anatomically defined compartment of functioning adenoma cells. These findings show that the benefits of MCSWR apply across different endocrine phenotypes, with the most notable improvements seen in tumors previously limited by hidden parasellar invasion.

These results represent a potential technical adjunct over both historical benchmarks and previous syntheses of this technique. Our pooled remission rate of 85% is markedly higher than the 63.3% reported in the 2023 meta-analysis by Pontes et al., a difference that likely results from including several additional, more recent series focused on functioning adenomas [11, 31–34] and the maturation of the procedure in high-volume centers using standardized ligamentous landmarks and a medial-to-lateral dissection strategy [13]. Furthermore, compared with standard TSS for invasive (Knosp 3–4) adenomas, which have historically had remission rates of 20% to 40%, MCSWR was associated with higher reported ER rates than historical benchmarks [17, 23, 24]. Although the convergence of radiographic GTR (92%) and ER (85%) is encouraging, the predominantly non-comparative data do not establish that MCSWR itself is responsible for these favorable outcomes, which may also reflect patient selection, surgeon experience, and institutional expertise. Likewise, the pooled recurrence rate of 6% should be interpreted cautiously given the relatively short and heterogeneous follow-up across contributing studies.

Despite the technical challenges of MCSWR, the pooled complication profile in this analysis was favorable and comparable to that of other cavernous sinus–targeted approaches. The lack of ICA injury in 481 reported cases (95% CI 0–1.0%) is especially notable, considering that major arterial injury rates in extended endonasal skull base surgery typically range from 0.1% to 4.2% [25]. This safety margin likely stems from the medial-to-lateral dissection approach, which keeps the surgical plane along the dural surface rather than the arterial adventitia, and from the consistent identification of ligamentous landmarks that define the operative pathway [12, 19]. Routine Doppler ultrasonography further enhances safety by providing real-time mapping of the ICA course, thereby reducing the risk of blind or misdirected dissection [26]. Cranial nerve morbidity was also low, with transient CN III and VI palsies occurring in about 3% of patients, rates that remain significantly lower than those reported for wider transcavernous or interdural approaches [12]. The pooled CSF leak rate of 4% aligns with current endoscopic pituitary series, which commonly use multilayer skull-base reconstruction, typically combining fat and/or fascia, and, when indicated, a pedicled nasoseptal flap, to reduce postoperative complications [27]. Overall, these findings suggest that, when performed in high-volume centers with standardized anatomical workflows, MCSWR has a safety profile comparable to or better than that of other parasellar treatments, supporting its broader use for appropriately selected patients.

Identifying which patients may benefit most from MCSWR remains essential, as medial wall resection should be targeted rather than routine. Accordingly, the clinical rationale for MCSWR is not removal of the medial wall per se, but the selective identification and resection of occult medial wall invasion in patients with suspected cavernous sinus involvement, including cases in which microscopic invasion cannot be definitively established radiologically or intraoperatively. Data from histologic and intraoperative studies show that microscopic medial wall invasion occurs even in tumors with low radiographic Knosp grades, with Ishida et al. finding histologic invasion in 57% of cases initially deemed non-invasive [15]. This finding, combined with the fact that the absence of a pseudocapsule has been associated with dural infiltration, suggests that radiographic Knosp grade alone may not fully capture the risk of medial wall involvement [28]. Endocrine phenotype may further inform patient selection: somatotroph and corticotroph tumors have been reported to involve the medial wall early in their natural course [21, 29], while dopamine-agonist–treated prolactinomas may develop dense fibrosis, which has been associated with more challenging surgery and lower rates of ER [30]. Conversely, true Knosp 4 encasement with circumferential ICA involvement should be approached cautiously, as invasion beyond the medial wall into the venous or interdural compartments may limit the safety of extracapsular dissection [12]. Therefore, potential candidates may include functioning adenomas that lack a clear pseudocapsule, DA-resistant prolactinomas with suspected fibrosis, and invasive tumors where medial wall involvement seems to contribute to biochemical persistence. When used within these parameters and performed at high-volume centers with expertise in ligament-based dissection and Doppler guidance, MCSWR may offer a useful adjunct for achieving biochemical remission while maintaining an acceptable safety profile in selected patients.

### Limitations

First, most of the included studies were single-arm retrospective series without direct comparator groups, which introduces selection bias and prevents head-to-head comparisons with conventional transsphenoidal surgery without medial cavernous wall resection. It also limits the generalizability of the favorable safety profile, including the 0% ICA injury rate, to lower-volume programs or surgeons who are earlier in their learning curve. Second, although we harmonized endocrine endpoints by adopting author-defined remission criteria aligned with current consensus guidelines, substantial heterogeneity remained not only in biochemical thresholds, imaging protocols, and follow-up durations, but also in the clinical indications for MCSWR, the extent of medial wall resection, Knosp grade, and the degree of intrasinus involvement. These differences limit direct between-study comparisons and make it difficult to determine which patients are most likely to derive benefit from the procedure. Third, sample sizes for specific subgroups, especially prolactinomas and ACTH-secreting tumors, were small, reducing statistical power. The risk-of-bias assessment using the ROBINS-I tool showed that most studies had a serious or critical risk of bias, mainly due to confounding and the lack of prospective control groups, as medial wall resection is often reserved for more invasive disease. Finally, the lack of very long-term follow-up (e.g., over 10 years) in many studies limits the ability to draw definitive conclusions about late recurrence, especially in CD. Although systematic histological assessment of resected medial wall tissue would strengthen future studies, the absence of histological confirmation in every case does not exclude occult invasion, as microscopic medial wall involvement may not be reliably predicted radiologically or intraoperatively.

## Conclusion

In this systematic review and meta-analysis, MCSWR for functioning pituitary adenomas was associated with high reported rates of endocrinological remission and gross total resection, and with low reported rates of recurrence and perioperative morbidity in selected cohorts. These findings support the medial cavernous sinus wall as a clinically relevant site for potential microscopic invasion and suggest that its selective resection may be a useful adjunct in carefully selected patients.

However, the current evidence remains limited by predominantly retrospective, single-arm studies, short and heterogeneous follow-up, inconsistent histological confirmation of medial wall invasion, variable reporting of cavernous sinus involvement, and a concentration of cases at high-volume centers. Therefore, these data should not be interpreted as definitive proof that MCSWR is superior to standard transsphenoidal surgery or that it should be routinely adopted for all functioning adenomas. Future prospective, multicenter studies with standardized endocrine endpoints, detailed reporting of Knosp grade and intrasinus extension, routine histopathological assessment of resected medial wall tissue, and long-term follow-up are needed to define the true incremental benefit, durability, and generalizability of MCSWR.

## Data Availability

Requests for the data used in this project can be obtained by emailing the corresponding author.
